# Exploration of the beliefs and experiences of Aboriginal people with cancer in Western Australia: a methodology to acknowledge cultural difference and build understanding

**DOI:** 10.1186/1471-2288-9-60

**Published:** 2009-08-13

**Authors:** Shaouli Shahid, Dawn Bessarab, Peter Howat, Sandra C Thompson

**Affiliations:** 1 Centre for International Health, Curtin University of Technology, GPO Box U1987, Perth WA 6845, Australia; 2 Centre for Behavioural Research in Cancer Control, Curtin University of Technology, Perth, Australia

## Abstract

**Background:**

Aboriginal Australians experience poorer outcomes, and are 2.5 times more likely to die from cancer than non-Aboriginal people, even after adjustment for stage of diagnosis, cancer treatment and comorbidities. They are also less likely to present early as a result of symptoms and to access treatment. Psycho-social factors affect Aboriginal people's willingness and ability to participate in cancer-related screening and treatment services, but little exploration of this has occurred within Australia to date. The current research adopted a phenomenological qualitative approach to understand and explore the lived experiences of Aboriginal Australians with cancer and their beliefs and understanding around this disease in Western Australia (WA). This paper details considerations in the design and process of conducting the research.

**Methods/Design:**

The National Health and Medical Research Council (NHMRC) guidelines for ethical conduct of Aboriginal research were followed. Researchers acknowledged the past negative experiences of Aboriginal people with research and were keen to build trust and relationships prior to conducting research with them. Thirty in-depth interviews with Aboriginal people affected by cancer and twenty with health service providers were carried out in urban, rural and remote areas of WA. Interviews were audio-recorded, transcribed verbatim and coded independently by two researchers. NVivo7 software was used to assist data management and analysis. Participants' narratives were divided into broad categories to allow identification of key themes and discussed by the research team.

**Discussion and conclusion:**

Key issues specific to Aboriginal research include the need for the research process to be relationship-based, respectful, culturally appropriate and inclusive of Aboriginal people. Researchers are accountable to both participants and the wider community for reporting their findings and for research translation so that the research outcomes benefit the Aboriginal community. There are a number of factors that influence whether the desired level of engagement can be achieved in practice. These include the level of resourcing for the project and the researchers' efforts to ensure dissemination and research translation; and the capacity of the Aboriginal community to engage with research given other demands upon their time.

## Conceptualising the study

The profound health disparities that arise out of political, social, economic, educational and other disadvantage experienced by Aboriginal and Torres Strait Islander (hereafter Aboriginal) people in postcolonial Australia are well described. In this paper, the term Aboriginal denotes Indigenous people of Australia. We have used "Indigenous" to refer to common features that are identified across different Indigenous peoples. As data collection systems have improved, it has also been recognised that Aboriginal Australians have a higher incidence of some preventable, especially smoking-related malignancies compared to non-Aboriginal Australians and are less likely to access cancer screening, more likely to be diagnosed at a more advanced stage and to have poor continuity of care and lower compliance with treatment[1]. Aboriginal cancer rates appear to be increasing and Aboriginal people experience lower survival for all cancers when adjusted for stage at diagnosis [1-3]. Further research that merely continues to describe these gaps is limited in its use.

In public health research, consideration should be given to both quantitative *and qualitative *research when planning health promotion interventions. Qualitative research is increasingly recognised as playing a role in understanding the determinants of health behaviour, and informing alternative approaches[4]. Qualitative research can also enable an appreciation of the socio-cultural and historical context in which the problems or risks are constructed[5] and provide information upon which specific interventions or changes in policy and practice can be based.

This research aimed to explore Western Australian (WA) Aboriginal perspectives and experiences of cancer, cancer services and treatment from the lived experience of Aboriginal people. The project was conceived to find out information which might assist the development of effective health promotion interventions in cancer control in the Aboriginal community.

This paper explores methodological considerations in conducting the research which was initiated by non-Aboriginal researchers in response to a need identified by service providers for greater understanding of Aboriginal beliefs about cancer[6].

## Key considerations and challenges in Indigenous research

Aboriginal and Torres Strait Islander people are the original inhabitants and the Indigenous people of Australia with a history of 60,000 years of habitation. Since the arrival of the first colonists in 1788 Aboriginal people's lives have changed dramatically. Devastating affects of colonization resound through the generations and is considered to be one of the underlying reasons impacting on the poor health of Aboriginal people today.

### Colonisation and decolonisation

It is imperative to understand the historical context of Aboriginal people in Australia when attempting to conduct research with Aboriginal communities and individuals. Part of the colonial legacy is the negative view that many Aboriginal people hold of research. This is a consequence of past unethical practices where research was inappropriately carried out *on *Aboriginal Australians rather than *with *them and was often undertaken without adequate consultation or informed consent[7,8]. Denzin and Lincoln (2008) have framed it this way – "Western scientists discovered, extracted, appropriated, commodified, and distributed knowledge about the Indigenous other[9]." All these experiences have contributed to research becoming a dirty word[10] to Indigenous communities with research linked to colonisation, oppression[11] and the exercise of power and control over Indigenous peoples. To minimize such consequences, scholars involved in Indigenous research have taken steps to make the research processes decolonized, ethical, responsible, accountable to and participatory for Indigenous peoples. There are now attempts to centralise Indigenous concerns, worldviews and perceived needs within research, to know and understand theory and research from their perspectives, and to better utilise the findings for their own benefit. This whole process is described as 'decolonisation' by Linda Tuhiwai Smith [10].

### Indigenous paradigm

In the last 20 years [12], there has been a paradigm shift in Australia and internationally when undertaking Indigenous research away from research which conceptualised and understood systems of knowledge in conventional positivistic social science terms. 'Indigenous scholars from Australia, Aotearoa-New Zealand, the United States, and Canada have brought to academic discussions the Indigenous peoples' project of reclaiming control over Indigenous ways of knowing and being'[11,13,14]. Indigenous methodological approaches are based on Indigenous epistemologies that privilege Indigenous voices and ways of knowing and understanding the social world. For Indigenous people, knowledge is relational and Indigenous knowledge systems are founded on relationships with other people, the land and everything around them. Indigenous epistemology recognizes that there is more than one reality and meaning in understanding the social world [15].

Whereas positivistic research is based on objectivity, qualitative research is subjective and undertakes to describe the social world through the lived experience of the participant. These two different research approaches lead to distinctly different research processes, design and methodology. The positivistic scientific research process requires researchers to remain outside the research experience, to investigate through observation and discovery, as objectively or neutrally as possible, and to draw conclusions based on those observations[11]. Indigenous research, on the other hand, develops a shared relationship between the researcher and the researched population who must be interconnected in a reciprocal way during the research process. Principles of respect, reciprocity and relationality are critical for Indigenous methodologies.

### Practical challenges and stigmatisation towards Aboriginal community

There can be difficulties in accessing Aboriginal people as research participants given that they are often marginalised, suspicious of research and of discussing personal experiences with strangers. In regional/remote areas, distance makes travel challenging and people difficult to access. Aboriginal people in communities often speak their own language as a first language with English being their second or third language. Cultural protocols and taboos must be followed when engaging with Aboriginal communities[7]; failure to do so can limit researchers' interaction with their Aboriginal participants and cause mistrust and misunderstanding. These factors may be some of the reasons behind the dearth of systematic investigation of what underlies poor Aboriginal cancer outcomes[1]. Apart from these access difficulties, negative stereotypes about Aboriginal Australians can influence non-Aboriginal people's attitudes and behaviours towards them[16] as it is unlikely that researchers are not influenced by stereotyping and the institutional and overt racism that exists in mainstream Australia.

## Axiology (values and ethics) in conducting this research

Indigenous axiology, which incorporates nature, types and criteria of values and value judgments, are of great importance in designing Indigenous methodologies; especially in relation to research ethics. Indigenous research ethics encourages researchers to incorporate alternative perspectives, and apply nuanced judgments to any ethical implications. Six values (see Table 1) outlined in the National Health and Medical Research Council (NHMRC) guidelines for ethical conduct of Aboriginal and Torres Strait Islander health research[17] in Australia, were followed for this particular study. Ethics approvals were obtained from the Human Research Ethics Committee (HREC) of Curtin University, the Western Australian Aboriginal Health Information and Ethics Committee, and the Royal Perth and Sir Charles Gairdner Hospitals. Approval was also obtained from local Aboriginal Health Services.

**Table 1:** The six key values that lie at the heart of research engagement with Aboriginal communities activities as recommended by the National Health and Medical Research Council and the activities undertaken to address those values while conducting this research.

**i. Reciprocity** Reciprocity entails the inclusion and recognition of participants' contributions and in return the delivery of research outcomes that benefit the communities or individuals. The benefit should be valued by Aboriginal individuals and communities.	• Sharing of knowledge and expertise • Assistance give to ARG members with their personal and professional needs • Researchers assisted with writing applications to gain support for ARG members • Funding for reimbursing some organisations that provided assistance • Capacity building as part of the research, e.g. through co-presenting research findings and assisting develop an Indigenous cancer support group as an outcome of the research
	
**ii Respect** Respect for individual and collective culture and acknowledgement of the right of Indigenous Australians to have different values, norms and aspirations are critical to the research process. This is fundamental to have a sustainable research relationship between participants and researchers.	• Consultation with and involvement of Aboriginal people throughout the research; guidance by ARG • Flexibility of the research design with modifications to reflect feedback • Acknowledgement of differing cultural beliefs and understanding of health and illness • Responsiveness to feedback and ensuring that some feedback of study results occurs to Aboriginal people
	
**iii Equality** Equality affirms Aboriginal and Torres Strait Islander peoples' right to be different and thus entails the appreciation and respect towards these differences while performing research.	• Valuing of Aboriginal knowledge and wisdom through exploring Aboriginal perspectives, knowledge and preferences to inform the research process • Actively encouraging Aboriginal involvement and support • ARG's comments and suggestions on any aspect of the project valued • Aboriginal people are co-presenters and co-authors of findings
	
**iv Responsibility** The recognition of "core responsibilities", including those to country, family, community and maintaining harmony between the spiritual and physical realms.	• Attention to minimizing risk and ensuring no harm to participants and no unintended consequences • Accountability to Aboriginal stakeholders • The research process included: adequate, transparent consultation, opportunities for feedback during the development and conduct of the research, distribution of research findings in a way that was accurately represented, appropriate and understandable
	
**v. Survival and Protection** The need to protect Aboriginal cultures from erosion and maintain the collective identity.	• Reflected in the aim of the research to explore Aboriginal perspectives and the intent of reporting the findings in a way that is respectful of Aboriginal values and does not inadvertently contribute to discrimination or derision of Aboriginal Australians
	
**vi Spirit and Integrity** It refers to show respect for the richness and diversity of Aboriginal and Torres Strait Islander peoples' cultural inheritance of past, present and future generations and of the links which bind the generations together and requires the behavioural and perceived integrity of the researchers.	• Recruitment strategy to capture a broad range of Aboriginal perspectives, enriching the diversity of knowledge obtained. • Flexibility around timeframes, recognition of the importance of relationships while conducting the research

Another important consideration is the nature of 'insider' and 'outsider' positions of research conducted in Indigenous settings[10]. Because the research team was university educated, predominately non-Aboriginal and not local community members, the researchers were aware they would be considered 'outsiders'. They were cautious about their interpretation of different issues while collecting data as they understood that they might not have the intimate, intuitive understanding of the world of an 'insider'. To assist with overcoming this, a local trusted person was engaged to introduce the interviewer on each rural and remote visit; sometimes they remained throughout the interview. This process assisted fostering the development of a trusting relationship with the participants and assisted the researchers to maintain local cultural protocols. Aboriginal participants were assured that they need only provide information that they were comfortable with sharing. Standard research processes were adopted to ensure the confidentiality of individuals and the integrity of the data collected.

## Research methodology

### Research design

This research was considered exploratory since few studies have examined Aboriginal peoples' understanding, knowledge and beliefs about cancer and experiences of cancer care[1]. The diversity of the Aboriginal population in Australia, to which further differences have accumulated in terms of acculturation, education and opportunity, meant that considerable variation was expected between participants. Two key concepts – 'meanings of cancer' and 'experiences with cancer and cancer services' were explored from the lived experiences of individuals. The phenomenological qualitative approach was chosen because of its suitability for research that seeks to provide an insight into how people make sense of, describe and interpret their experiences and portray the process involved in a phenomenon. This methodology aims to extract "the contextualized nature of experience and action, and attempts to generate meaning that are detailed, 'thick', and integrative[18]."

The establishment of an Aboriginal Reference Group (ARG) at the beginning of the research was crucial to assist and ensure that all stages of the research adhered to and acknowledged community values and aspirations. Members of the ARG were acknowledged as professionals both by their Aboriginal and non-Aboriginal peers in the areas of Aboriginal health and welfare. Initially, researchers utilized their personal networks to identify Aboriginal people who were working in different cancer services and in other Aboriginal health services in WA. Researchers then approached them personally, explained the initial research plan, processes and the purpose of forming the ARG. Aboriginal people, who expressed interest, to the extent of being willing to commit their time were formally requested to be a part of the ARG. The management of data, the protection of individual and community identity and the dissemination of findings were discussed throughout with the ARG members.

### Development of an interview guide

An open-ended, exploratory general theme list that could guide semi-structured interviews was initially developed. The guide was based upon an in-depth examination of common themes identified in the existing literature on cancer beliefs and understanding among the Indigenous population in Australia, Canada, New Zealand and the USA[1]. This literature guided the approach and the design of an appropriate qualitative research instrument, the approach to data collection and the analysis in the subsequent phases of the research. The initial draft was modified after discussion with the ARG. The topic list is included in the Appendix.

### Participant recruitment and data collection

Data collection occurred in two rural and one remote community and in the urban Perth metropolitan area. Thirty interviews were conducted among Aboriginal cancer patients, survivors and family members of people with cancer or who had died of cancer. Interviewees were male and female adults. Recruitment initially occurred through the networks of the researchers and reference group and also through health professionals in primary or tertiary care. Some limited snowball recruitment occurred as initial participants recommended others as candidates for the study, but care was taken to ensure a mix of males and females, types of cancer and geographical locations. Recruitment and data collection continued until the research team was satisfied the data was comprehensive and rich, and there was repetition of themes in the interviews with new participants[18].

Agreement for the research to occur was obtained from local community leaders and community health organisations in rural or remote regions. Whenever possible, participants were given an information statement about the research well before the interview which clearly explained its purpose, procedures, risks and benefits including the rights of the participant and contact information for the researcher. Participants were invited to have a support person present at the interview if they wished. Before beginning the research interview, time was spent building a relationship with the participant. Several strategies were used, for example, the interviewer visiting several times before the actual interview date, or sharing her personal stories and background to make the participant feel comfortable and to assist with establishing a relationship. The interviewer explained the research and obtained written consent before the formal interview commenced; agreement for the interview to be recorded was obtained separately. Interviews varied considerably in length, commonly lasting around 1.5 hours.

Once the relationship had been established and any initial anxiety was reduced, participants were asked to share the story of their journey with cancer (either their own or their family member). They were asked to include their experiences of diagnosis, treatment, recurrence of cancer and to suggest strategies to address the issues they faced during their cancer journey. They were also asked about their perspectives and meaning attached to cancer. A narrative method was chosen because the study aimed to explore the complexity and in-process nature of meanings and interpretations of Aboriginal men and women's experience of cancer. The interviews were akin to conversations, letting the interviewees talk freely and frankly about their cancer experience, and the meanings and understandings they attributed to it. This flexible style was chosen so that people's voices could be heard accurately and in their own way. Some interviewees reported they had not previously discussed their cancer experience and many found it quite liberating, even cathartic, to have the opportunity to reflect on and talk about it. The topic list was not generally needed to guide the interviews.

Information was also collected from relevant Aboriginal and non-Aboriginal health service providers from a mixture of primary and tertiary health care service settings. Their inclusion ensured triangulation of views on Aboriginal perceptions and engagement with cancer-related services. It also helped to recognise the nature of differences that Aboriginal people and health service providers had in understanding cancer and relevant services. Twenty providers were interviewed between March 2006 and September 2007.

In addition, a field log was carefully maintained throughout, documenting impressions, observations, any incidents, the research process, and reflections upon limitations and negative events. These reflections were linked with the responses during data analysis.

### Data analysis

All interviews were audio-recorded and transcribed verbatim. Initially, manual open coding[19] was carried out independently by two researchers who carefully read and re-read hard copies of the transcript. Differences were later discussed and resolved in the research team[20]. Detailed coded texts were entered into N-Vivo7 software and the distribution of important codes was identified, highlighted and grouped according to major categories and sub-categories developed from the text and a background literature review[1]. Feedback sessions with available participants assisted clarification of whether emerging themes were an accurate reflection of participants' experiences.

Social constructivism, which incorporates a social ecological and holistic approach, was considered during the interpretation and analysis of data [21-23]. The application of social ecological frameworks examines the multiple effects and interrelatedness of several social elements[24] to establish a bigger picture in explaining a phenomenon. This framework explicitly recognises that "the well-being of the individual is predicated on the well-being of the immediate family, which, in turn, is contingent upon community and societal conditions[21,22]. This approach is well suited to Aboriginal health research because it aligns with Indigenous holistic values and considers physical, mental, emotional and spiritual aspects of healing and wellness together for the total well-being of an individual[23].

## Discussion

This paper describes the research approach and methods of a study exploring Aboriginal Australian beliefs and experiences around cancer and cancer services in WA (results are reported elsewhere[25]. The researchers considered and were respectful of key steps of conducting Indigenous research. However, there were limitations, some that could not be overcome. It was not possible, despite the efforts of the researchers, to secure funding beyond one year for the research, which was a major constraint upon providing the optimal means of feedback to participants and the Aboriginal community; and for research translation. However, to minimise the fact that the project did not get funding for research translation, the findings were presented to Aboriginal community representative forums organised by other organisations and their feedback obtained. Copies of the interviews were sent to some participants and permission was given to other organisations to utilise the study findings in their activities. However, this falls short of the personal feedback to study participants that would be optimal. The long time it takes for data collection, analysis and reporting is another reality for researchers that is not well understood by members of the Aboriginal community.

Funding constrained capacity in other ways; the main interviewer was female whereas ideally an Aboriginal male interviewer may have been important for recruiting male participants and their willingness to talk freely. Many of the ARG members were females, the community-based health workforce are overwhelmingly female, and females are known to attend health services more commonly than males, all of which are likely to have favoured recruitment of more females than males. Another challenge was that the researchers were often dependant on a local person to contact and liaise with the participants on their behalf, which sometimes limited the researchers' choices and opportunities.

The researchers were reliant upon the ARG as a conduit to the Aboriginal community. Coordinating the ARG as a group advisory network proved challenging due to members' individual commitments and work. Aboriginal professionals often have membership on several reference groups for different projects to be managed on top of their core job role, and this can place additional stress and pressure on them. Thus, when group meetings were difficult to convene, members of the group had to be contacted individually which was time consuming and provided input of a different nature to that of a face-to-face meeting.

The Indigenous research paradigm with its need for the research process to be relationship-based, respectful, culturally appropriate and inclusive of Aboriginal people challenged the training and experience in positivist social science of the interviewer. Building the trustworthy relationship with the participants before doing the actual interview created important insights during the research about the life of contemporary Aboriginal people and their concerns.

Presentations of the findings by the researchers needed to be tailored to the audience with the researchers being conscious of balancing their responsibility and obligation to their participants and the wider Aboriginal community with the academic expectations of their disciplines. Wherever possible, an Aboriginal co-presenter assisted with presentations. There are specific guidelines from journals concerning criteria for authorship and the desire to include Aboriginal authors must be balanced against tokenism. In the current study, authorship issues were given careful consideration and based upon a substantial contribution to the conception, conduct, analysis and writing of the research. One of the authors in this paper is an Aboriginal researcher who was involved with the research and has contributed significantly to publications arising from the study.

Given our commitment to working with the Aboriginal community, it is important to consider how they benefit from this research. Arising out of contacts made during data collection in one regional area, the researchers supported an Indigenous woman to establish an Indigenous Women's Cancer support group[26], and are continuing to work with the group around resourcing and developing a working partnership with mainstream services. There have been opportunities for capacity development of Indigenous people as researchers in the process including them undertaking university postgraduate coursework and research, as co-presenters during presentations in conferences, seminars and lectures and as co-authors on publications arising from this study. Given the dearth of understanding that service providers had of issues relevant to Aboriginal people and cancer, the systematic consideration of the understanding, views and experiences that Aboriginal people have with regard to cancer and that impact upon their access to cancer prevention and treatment services has been important. Information has been disseminated to prompt relevant agencies to improve health and social support in favour of the health and well-being of Aboriginal people. The information and advocacy efforts have influenced policy planners and service providers to acknowledge the need for approaches different to traditional mainstream services. The findings are also informing and assisting the development of appropriate messages with regard to cancer in Aboriginal communities.

Our approach has elements of community-based participatory research which is research conducted as an equal partnership between traditionally trained "experts" and members of a community, and is generally iterative in nature, incorporating research, reflection, and action in a cyclical process[27]. The nature of our research, the research funding constraints, and the many demands upon the small Indigenous population (both community members and health professionals), would create many challenges for truly equitable partnerships. Moreover, community-based participatory research is most likely to be effective in creating change if it arises in the community and has a clear intention to being action-oriented[28]. This seems most likely to be achieved if there is long-term engagement and adequate time and resourcing for each partner, requisites not overcome simply by good intentions.

Western researchers and academics are becoming more appreciative of the need to work with Indigenous researchers as part of decolonising research methodologies and, to incorporate appropriate processes in research with Indigenous people. Some of the key issues of Indigenous research methodology – including the need for being attentive to the culture and traditions of the population they are working with, the necessity to make the process participatory and inclusive of Indigenous communities, the requirement for providing feedback to the community – are equally applicable to other culturally distinct and marginalised communities in the world. However, the profound effect of colonisation on the Australian Indigenous population and its legacy of mistrust and suspicion has a huge impact which needs to be acknowledged and addressed in approaches to Indigenous research. The ongoing challenge is to prioritise responsible conduct of research that ensures a social justice outcome, builds the capacity and develops positive relationships with the researched populations, and creates spaces for Indigenous voices to be heard. This view has also been supported by researchers conducting Indigenous and cross-cultural research in other countries[29].

## Competing interests

The authors declare that they have no competing interests.

## Authors' contributions

SS participated in the project's design, carried out the data collection and analysis for this project, prepared the initial draft. DB was involved in writing. PH commented upon drafts of the manuscript. SCT coordinated the whole project, participated in the design and assisted with the conduct of the study and writing. All authors read and approved the final manuscript.

## Appendix

### Issues explored during interview

1. Journey with the illness

• Experiences with diagnosis, including causes of delayed diagnosis

• Treatment phase

• Coping with cancer

• Recurrence of cancer

• Death and dying

2. On services

• Experiences and issues with the hospitals/health services/cancer services

• Good experience/facilities

• Bad experiences/difficulties/problems with the health system

• How to address the problems they faced in the health services

• What is needed for cancer patients, and Aboriginal cancer patients

• What supports they got and from where

• Any barriers to screening, diagnosis or treatment

3. Perspectives of cancer

• What is cancer

• Causes and aetiology of cancer

• How to get rid of this illness?

• Impact – How it changes someone's life

• Meaning attached to cancer

## Pre-publication history

The pre-publication history for this paper can be accessed here:

http://www.biomedcentral.com/1471-2288/9/60/prepub

## References

[B1] Shahid S Thompson SC An overview of cancer and beliefs about the disease in Indigenous people of Australia, Canada, New Zealand and the United States Aust N Z J Public Health 2009 33 2 109 118 10.1111/j.1753-6405.2009.00355.x 19413852

[B2] Cunningham J Rumbold AR Zhang X Condon JR Incidence, aetiology, and outcomes of cancer in Indigenous peoples in Australia Lancet Oncol 2008 9 585 595 10.1016/S1470-2045(08)70150-5 18510990

[B3] Valery PC Coory M Stirling J Green AC Cancer diagnosis, treatment, and survival in Indigenous and non-Indigenous Australians: a matched cohort study Lancet 2006 367 1842 1848 10.1016/S0140-6736(06)68806-5 16753487

[B4] Newell S Sanson-Fisher RW Australian oncologists' self-reported knowledge and attitudes about non-traditional therapies used by cancer patients MJA 2000 172 110 113 10735020

[B5] Denzin NK Lincoln YS The Sage Handbook of Qualitative Research 2005 California: Sage Publications, Inc

[B6] Shahid S Beckmann KR Thompson SC Supporting Cancer Control for Indigenous Australians: Initiatives and Challenges for Cancer Councils in Australia Aust Health Rev 2008 32 56 65 18241149 10.1071/ah080056

[B7] Holmes W Stewart P Garrow A Anderson I Thorpe L Researching Aboriginal health: experience from a study of urban young people's health and well-being Soc Sci Med 2002 54 1267 1279 10.1016/S0277-9536(01)00095-8 11989962

[B8] Rigney LI Internationalisation of an Indigenous anti-colonial critique of research methodologies: A guide to Indigenist research methodology and its principles Journal for Native American Studies 1997 14 109 121

[B9] Denzin NK Smith LT Lincoln YS Denzin NK, Smith LT, Lincoln YS Introduction: critical methodologies and Indigenous inquiry Handbook of Critical and Indigenous Methodologies 2008 Sage Publications

[B10] Smith LT Decolonising methodologies: Research and Indigenous Peoples 1999 London & New York: Zed Books

[B11] Porsanger J An essay about Indigenous methodology Doctoral Seminar presentation 1999 University of Tromso http://uit.no/getfile.php?PageId=977&FileId=188

[B12] Rigney LI A first perspective of Indigenous Australian participation in science: framing Indigenous research towards Indigenous Australian intellectual sovereignty Kaurna Higher Education Journal 2001 1 13

[B13] Vicary D Bishop B Western psychotherapeutic practice: Engaging Aboriginal people in culturally appropriate and respectful ways Australian Psychologist 2005 40 8 19 10.1080/00050060512331317210

[B14] McKinley B Brayboy J Toward a tribal critical race theory in education The Urban Review 2005 37 425 446 10.1007/s11256-005-0018-y

[B15] Wilson S What is an Indigenous research methodology? Canadian Journal of Native Education 2001 25 175 179

[B16] Rowlands I Lee C Choosing to have children or choosing to be childfree: Australian students' attitudes towards the decisions of heterosexual and lesbian women Australian Psychologist 2006 41 55 59 10.1080/00050060500391860

[B17] NHMRC Values and ethics: Guidelines for ethical conduct in Aboriginal and Torres Strait Islander health research Canberra 2003

[B18] Liamputtong P Ezzy D Qualitative Research Methods 2005 Second South Melbourne, Victoria: Oxford University Press

[B19] Strauss A Corbin J Basics of qualitative research: Grounded theory, procedures and techniques 1998 Newbury Park, CA: Sage

[B20] Chenitz WC Swanson JM From practice to grounded theory: Qualitative research in nursing 1986 United States of America: Addison-Wesley Publishing Company

[B21] Fondacaro MR Weinberg D Concepts of social justice in community Psychology: Toward a social ecological epistemology Am J Community Psychol 2002 30 473 492 10.1023/A:1015803817117 12125778

[B22] Prilleltensky I Nelson G Doing Psychology critically: Making a difference in diverse settings 2002 London: Palgrave Macmillan

[B23] Aboriginal Cancer Care Unit Analysis of the findings: Aboriginal cancer care needs assessment, "It's our responsibility"... Volume 1 August, 2005 2002 Toronto: Aboriginal Cancer Care unit, Cancer Care Ontario

[B24] Oetzel JG Ting-Toomey S Rinderle S Oetzel JG, Ting-*Toomey S Conflict communication in contexts: a social ecological perspective The SAGE handbook of conflict communication 2006 Thousand Oaks, CA: Sage

[B25] Shahid S Finn L Thompson SC Barriers to participation of Aboriginal people in cancer care: communication in the hospital setting MJA 2009 190 574 579 10.4066/amj.2008.31 19450207

[B26] Finn L Pepper A Gregory P Thompson SC Improving Indigenous access to cancer screening and treatment services: descriptive findings and a preliminary report on the Midwest Indigenous Women's Cancer Support Group AMJ 2008 1 1 21 10.4066/amj.2008.31

[B27] Wallerstein N Duran B Wallerstein MMN The conceptual, historical and practice roots of community based participatory research and related participatory traditions Community based participatory research in health 2003 San Francisco: Jossey-Bass 27 52

[B28] Cook WK Integrating research and action: a systematic review of community-based participatory research to address health disparities in environmental and occupational health in the USA J Epidemiol Community Health 2008 62 668 676 10.1177/1077800408318304 18621950 PMC2772828

[B29] Lincoln YS Elsa M Gonzalez EMGy The search for emerging decolonizing methodologies in qualitative research Qualitative Inquiry 2008 14 784 805 10.1177/1077800408318304

